# Linking Mitochondrial Function to Insulin Resistance: Focusing on Comparing the Old and the Young

**DOI:** 10.3389/fnut.2022.892719

**Published:** 2022-06-23

**Authors:** Jingxuan Wang, Junnan Wu, Wenjing Li, Xinyu Wang, Ruifang Liu, Tao Liu, Jianhua Xiao

**Affiliations:** ^1^Key Laboratory for Prevention and Control of Common Animal Diseases in General Higher Education Institutions of Heilongjiang Province, College of Veterinary Medicine, Northeast Agricultural University, Harbin, China; ^2^College of Veterinary Medicine, Northeast Agricultural University, Harbin, China

**Keywords:** insulin resistance, high-fat diet, diabetes, young, elderly, rats

## Abstract

Long-term intake of high-energy diet can lead to decreased insulin sensitivity and even insulin resistance, eventually leading to diabetes. Diabetes often occurs in middle-aged and elderly people. However, there is growing evidence that the incidence rate of young body is increasing over the years. This means that insulin resistance can be caused by excessive energy intake in both young and old people. In this study, high-fat diet (HFD) and normal diet were fed to rats of elderly experimental group (EE), elderly control group (EC), young experimental group (YE), and young control group (YC), respectively, for 8 weeks, by which insulin resistance model was obtained. Insulin sensitivity was measured, histopathology changes in liver and skeletal muscle tissues were observed, and mitochondrial fusion and division and cell senescence were detected in four groups of rats. The results showed that both young and elderly rats developed significant insulin resistance, fat deposition, decline of mitochondrial function and mitochondrial biosynthesis in liver and skeletal muscle, and cell aging after HFD feeding. In addition, the degree of mitochondrial dysfunction and aging in young rats was similar to that of aged rats fed a normal diet after HFD. This experiment provides a reference for an in-depth study of the regulatory mechanisms of cellular energy metabolism in this state.

## Introduction

With the improvement of living standards, the incidence of diabetes has also increased, and it has gradually become one of the most important non-communicable diseases threatening global human health. Insulin resistance diseases caused by obesity and overnutrition are increasingly common in humans and animals (1–3). Currently, there are also increasing rates of obesity and insulin resistance in animals around the world. Insulin resistance is the cause of obesity, type 2 diabetes mellitus (T2DM), and other metabolic diseases (4, 5). Insulin plays an important role in glucose homeostasis and is the main regulator of carbohydrate, fat, and protein metabolism (6, 7). *In vivo*, insulin acts in liver, skeletal muscle, and fat by binding to insulin receptors therein (8). When a high-fat diet is fed for a long time, in order to promote the uptake of this glucose by cells, the body compensatory secretes too much insulin, which reduces the sensitivity of liver and skeletal muscle cells to insulin, leading to the occurrence of insulin resistance, which in turn leads to diabetes.

The prevalence of obesity and type 2 diabetes in animals will increase significantly with age, due to which aging will lead to a progressive decline in most endocrine functions and meanwhile result in severe metabolic disorder (9). Previous studies have shown that the prevalence of diabetes in the elderly is high, but at present, many studies show significantly lower insulin sensitivity in obese young adults (10). Young people with T2DM or abnormal glucose tolerance have lower βcell sensitivity than healthy old people, which may be related to the great demand for a role of β cells in regulating blood glucose in young people (11). It is worth noting that the incidence of diabetes in puppies and kittens has been increasing in recent years (12–14). At present, there is no report on young animals.

Growing evidence suggests that insulin resistance caused by high-energy diet can occur in middle-aged and elderly or even young animal individuals, by disturbing a variety of metabolic pathways in the body, which in turn leads to the occurrence of metabolic diseases such as obesity and T2DM. However, several reports have raised the question of whether young and old IR mechanisms are the same (15–17). Therefore, in this experiment, rats will be used as experimental animals to establish a high-energy diet-induced insulin resistance model for youth and old rats, based on which the effects of high-energy diet on the occurrence of insulin resistance, liver, and skeletal muscle as well as mitochondrial function in young and elderly rats will be elucidated, which will have a positive effect on the prevention of energy excess metabolic diseases.

## Materials and Methods

### Animals

The *in vivo* experiment was performed in accordance with The Tab of Laboratory Animal Welfare and Ethics Committee of Northeast Agricultural University. Twenty 3-month-old (300 ± 20 g) and twenty 15-month-old (500 ± 40 g) Sprague-Dawley (SD) male rats were purchased from Liaoning Changsheng Biotechnology Co., Ltd. (China) and housed under 12-h light/ dark cycle, controlled humidity (40 ± 10%), and constant temperature (24 ± 1°C).

After 1wk of acclimatization, rats of two ages were randomly assigned into four groups (*n* = 10, 5 rats per 549 × 395 × 200 mm cage).

### Experimental Design

**Figure 1:**
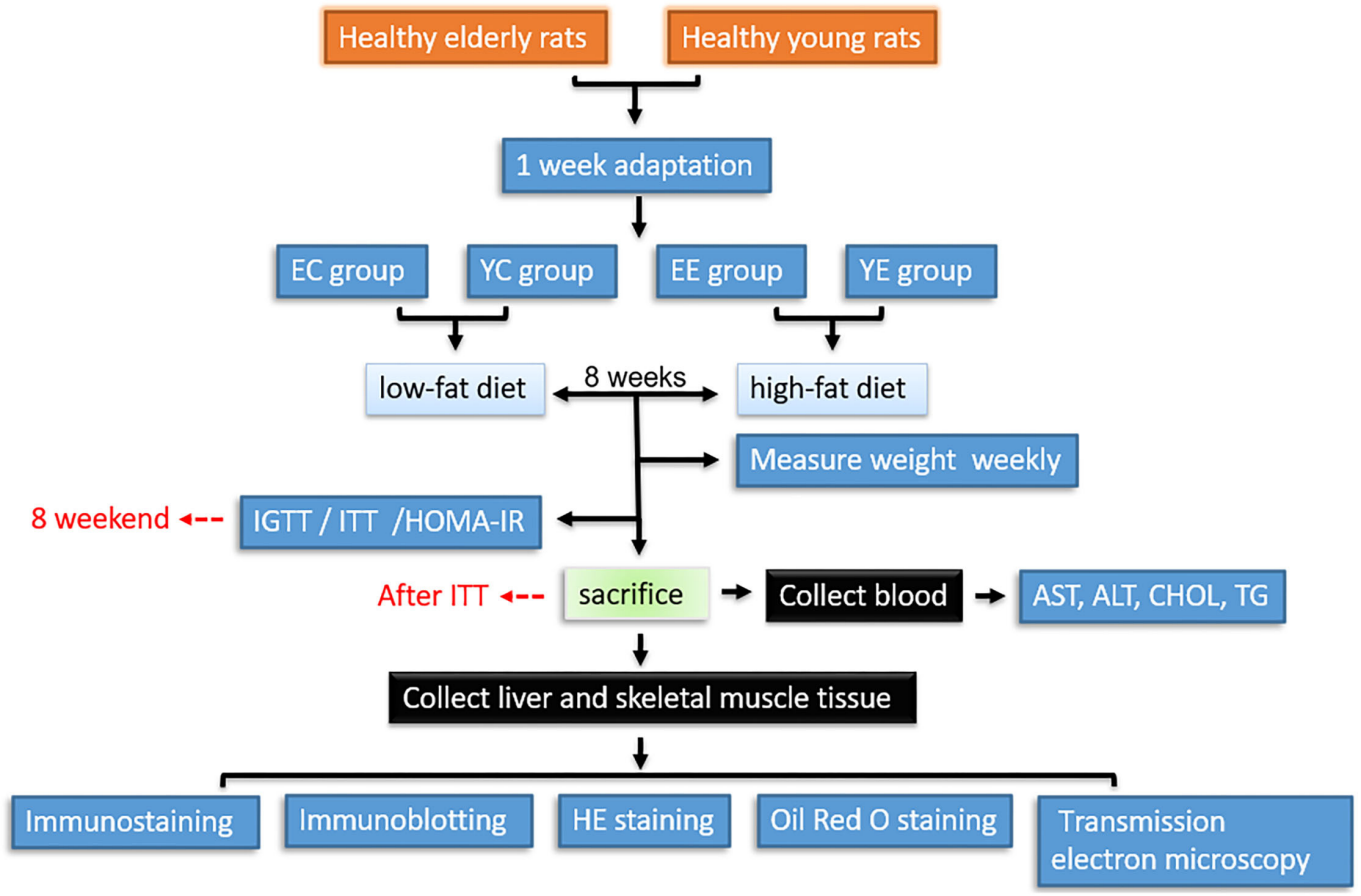
Workflow for hematology, histopathological, and proteomic analysis of tissues from insulin resistance rats. YC, young control group; YE, young experimental group; EC, elderly control group; EE, elderly experimental group.

After a week adaptation period, rats were allocated into four groups (*n* = 10 per group) as follows: (1) elderly experimental group (EE), with high-fat diet; (2) elderly control group (EC), with low-fat diet; (3) young experimental group (YE), with high-fat diet; (4) young control group (YC), with low-fat diet. All animals received water ad libitum, and all rats were maintained for 8 weeks. Glucose tolerance test (IGTT) was performed at the end of week 8, followed by insulin tolerance test (ITT) on day 3, that is, at week 9, rats were euthanized and blood samples were collected by cardiac puncture. Tissues were then harvested. Sera were separated and stored at −20°C for measuring biochemical parameters. Body weight of all rats was measured weekly between 8:00 and 9:00h (Figure 1).

Rats were divided into four groups: The 45% high-fat diet (D12451) was purchased from Research Diets Inc (New Brunswick, NJ, USA). The 4% fat rat-chow diet (CS101) was purchased from Liaoning Changsheng Biotechnology Co., Ltd. (Liaoning, China). The composition of the diets is shown in Table 1.

**Table 1:** Ingredient composition of the diets fed to rats (g/kg).

**Ingredient**	**4% fat**	**Ingredient**	**45% fat**
	**g/kg diet**		**g/kg diet**
Crude ash	80	Casein, 80 Mesh	200
Fiber	50	L-Cystine	3
Mineral mixture	30.215	Corn starch	72.8
Vitamin mixture	318.04	Maltodextrin 10	100
Lysine	8.2	Sucrose	172.8
Methionine + Cystine	5.3	Cellulose, BW200	50
Arginine	9.9	Soybean oil	25
Histidine	4	Lard*	177.5
		Mineral mix S10026	10
		DiCalcium Phosphate	13
		Calcium Carbonate	5.5
		Potassium Citratr, 1 H2O	16.5
		Vitamin Mix V10001	10
		Choline Bitartrate	2
		FD&C Red Dye #40	0.05
Protein kcal (%)	18	Protein kcal (%)	20
Fat kcal (%)	4	Fat kcal (%)	45
		Carbohydrate kcal (%)	35

*Typical analysis of cholesterol in lard = 0.95 mg/gram.

### Establishment of Insulin Resistance Model

#### Intraperitoneal Glucose Tolerance Test (IGTT)

Intraperitoneal glucose tolerance test was conducted at the end of the 8th week. Rats fasted overnight, and the baseline blood glucose level was measured using a blood glucose monitor (Sinocare, Hunan, China) via tail nick. Animals were injected intraperitoneally with 50% glucose (2 g/kg). Blood glucose levels were then measured at 0, 15, 30, 60, and 120 min after injection. The incremental area under the curve (AUC) of glucose response was calculated using GraphPad Prism 7.0 Software. The blood glucose concentration at 0 min was the fasting blood glucose (FBG, mmol/L) of rats.

#### Insulin Tolerance Tests (ITT)

After the glucose tolerance experiment, the rats recovered for 3 days. Rats in each group were fasted but not prohibited water for 12 h. Short-acting insulin (0.4 ml/kg body weight; Novo Nordisk, Gentofte) was administered intraperitoneally (i.p.) to rats, and blood samples were taken from the tail vein of the conscious rats before and 15, 30, 60, and 120 min after insulin administration (18–20). Blood glucose levels were immediately measured with a blood glucose monitor (Sinocare, Hunan, China). Total AUC was calculated by the GraphPad Prism 7.0 Software for 120 min after glucose injection.

#### Homeostatic Model Assessment of Insulin Resistance

The fasting insulin was measured using the ultrasensitive rat insulin enzyme-linked immunosorbent assay kit, and homeostatic model assessment of insulin resistance (HOMA-IR) was calculated as follows:


$$\begin{array}{l} \left[fasting\;glucose\;\left(mmol/L\right)\;\times \;fasting\;insulin\;level\;\left(\mu IU/mL\right)\right]/22.5 \end{array}$$

The homeostasis model assessment (HOMA) uses the product of basal insulin and glucose concentrations divided by a constant (21).

### Hematology Analysis

#### Blood Collection

After the ITT, the animals were killed. Blood samples were collected from the animals using cardiac puncture and put into different precooled EDTA containers. The blood samples were centrifuged at 4°C, 2,500 rpm for 15 min to obtain plasma. Each of the plasma was aspirated into plain sample bottles and stored at −80°C freezer until ready for biochemical analysis.

### Biochemical Analysis

Liver enzymes (AST, ALT, CHOL, and TC) in the plasma were analyzed with Mindray BS-280 automatic biochemistry analyzer (BS-280) (Nanjing Baden Medical, Jiangsu, China).

### Histopathological Examinations

#### Tissue Preparation and Histological Analysis

A portion of liver and skeletal muscle tissues were dissected and fixed with 4% paraformaldehyde for 72 h. The specimens were dehydrated in ascending grade of alcohols and paraffin embedding using standard methods. Then, the specimens were then sectioned in sagittal plane with 4-μm thickness and were stained with hematoxylin–eosin (H&E) staining analyzed to elucidate the status of tissue architecture and pathological analysis. Microscopic images were acquired using light microscopy.

The other part was kept frozen in a −80°C freezer for immunoblotting analysis and for making cryosections for Oil Red O staining. Liver and skeletal samples were fixed in 4% buffered formalin. Liver samples were embedded in optimal cutting temperature (OCT) medium and stored at −80°C. OCT embedded, 7 μm sections were stained with ORO for fat content examination. Randomly chosen areas of tissue sections were photographed using a light microscope.

#### Muscle Triglyceride Analysis

After dissecting any visible adipose tissue, 50 mg of tissue of each animal was weighed and homogenized in a handheld tissue homogenizer, using a volume of distilled water equal to eight times the tissue weight in mg. The protocol for triglyceride extraction was as follows: 5 M NaCl, methanol, and chloroform were added to the homogenate and mixed, and the ternary phase was broken with water and chloroform after 5 min of incubation. The three-phase system was then separated by centrifugation at top speed. The aqueous and protein phases were re-extracted with a 9: 1 chloroform: methanol wash solvent and separated by centrifugation. The organic solvent was dried using a stream of nitrogen gas with an N-EVAP, and the triglyceride was resuspended in isopropanol containing 2% Triton X-100. Glycerol concentration was measured relative to a glycerol standard curve. Standards and samples were loaded on a 96-well-plate, and free glycerol reagent was added to each well. The plate was incubated at 37°C and read on a Kinetic Microplate Reader at 540 nm. Triglyceride reagent was added to each well and incubated at 37°C. The plate was read again on the microplate reader at 540 nm, and the background glycerol was subtracted.

#### Muscle Glycogen Test

Using anthrone method (the kit is provided by Nanjing Jiancheng biology Co., Ltd. Article No.: A043), the test is carried out with method 721 spectrophotometer. Then, take 100 mg fresh muscle, and after treatment, mix the tissue with 300 μL alkali liquor (i.e., sample weight: alkali liquor volume = 1:3) into the test tube, boil with boiling water for 20 min, cool with running water, operate in strict accordance with the instructions of the kit, and substitute it into the formula to calculate the value.

#### Liver ATP Content Test

All reagents for ATP determination were prepared with redistilled distilled water. The solutions were as follows: (1) adenylate extract (Tris-HCL 20 mmol/L, MgSO_4_ 2 mmol/l); (2) luciferase buffer: each powder was used to dissolve in 50 mL of redistilled water, containing 50 mmol/L glycylglycine (PH=7.6), 10 mmol/L MgSO_4_, and 1 mmol/L EDTA buffer.

Standard curve of ATP: ATP was prepared into six tubes of application solution of *1*× *10*^−10^*-5*× *10*^−5^ *mol/L* for determination, and the standard curve was drawn by the *log* value of relative luminescence intensity and ATP concentration.

ATP determination by luciferase-luciferin method: Take 0.1–0.15 g of liver tissue, add it to 1 mL of adenylate extract, homogenize and heat in boiling water for 3 min, then centrifuge at 4,000 rcf/min for 3 min, and take 0.4 mL of supernatant. During detection, add 0.1 mL of liver homogenate to 0.1 mL of double distilled water to dilute, put it into a 2 mL cuvette, put it into the darkroom of FG-200 luminometer, and quickly inject 0.8 mL of luciferase-based buffer into the small hole of the dark chamber cover, and the initial peak of the recorded luminescence curve is the light intensity of the detected sample. The measurement temperature is 25°C, the measurement voltage is 0.5 mV, and the ATP value is detected on the standard curve according to the obtained light intensity (the number of small cells occupied on the measurement curve).

#### Observation of Mitochondrial Ultrastructure

The ultrastructure of liver mitochondria was observed with transmission electron microscopy (TEM). Pieces of liver tissue (1 mm^2^) were picked out within 1 min of death and fixed with 4% glutaraldehyde. Then, the samples were post-fixed in 2% osmium tetroxide, dehydrated in an ascending series of ethanols, and embedded in araldite. Ultrathin sections were cut with an LKB-8800 ultramicrotome (LKB, Sweden) and collected on grids. Sections were stained with uranyl acetate and lead citrate and evaluated with an H-500TEM (Japan) operated at 75 kV.

#### Western Blot

Samples containing 50 μg proteins were subjected to 10% SDS–PAGE and transferred to a nitrocellulose membrane. Membranes were blocked at room temperature for 2 h in blocking buffer containing 5% non-fat dry milk to prevent non-specific binding and then incubated with primary antibodies overnight at 4°C. The primary antibodies used in this study were GAPDH (1:750), anti-AMPK (1:3,000), anti-Mfn2 (1:5,000), anti-Opa1 (1:3,000), anti-Drp1 (1:1,500), anti-PGCα (1:1,500), anti-p53 (1:750), anti-p21 (1:1,000), and anti-p16 (1:5,000) antibodies. The membranes were washed in 50 mmol/L Tris–HCl, pH 7.6, 150 mmol/L NaCl, and 0.1% Tween 20 for 30 min and incubated with appropriate secondary antibody (1:3,000) for 2 h at room temperature. The nitrocellulose membrane was visualized using an ECL luminescent solution, and the film was exposed and visualized and photographed by a fully automated WB chemiluminescence imaging system (Tanon 5200, Shanghai Tanon Technology, China).

#### Immunostaining

The postfixed specimens of the liver were embedded in paraffin according to standard protocols. Paraffin-embedded sections (4 um) were preheated at 37°C for 10min. followed by incubation in xylene and stepwise rehydration in 100, 95, 70, and 50% ethanol. After slides were washed in TBS, the sections were blocked by the addition of normal goat serum diluted in TBS for 1 h at room temperature. Affinity-purified antibody to Opa1, Mfn2, Drp1, AMPK, PGC-1a, p53, p21, and p16 diluted in blocking solution was added at concentration of 2 ug/ml for 1h at room temperature followed by three 3-min washes with TBS at RT. Antirabbit IgG was added to the sections for 1 h at room temperature followed by three 3-min washes in TBS at room temperature and kept at 4°C until visualized.

### Statistical Analysis

The statistics and analysis of all data in this experiment were plotted using GraphPad Prism 8 statistical analysis, and the values were expressed as mean ± standard deviation (Mean ± SD). One-way analysis of variance was used to analyze the differences, *P* < 0.05 indicated significant differences, and ^*^ indicated differences. *P* < 0.01 indicated extremely significant difference, which was expressed as ^**^. *P* > 0.05 indicates no significant difference, denoted as *ns*.

## Results

### Basic Characteristics of the Experimental Animals

#### Appearance and Body Weight

**Figure 2:**
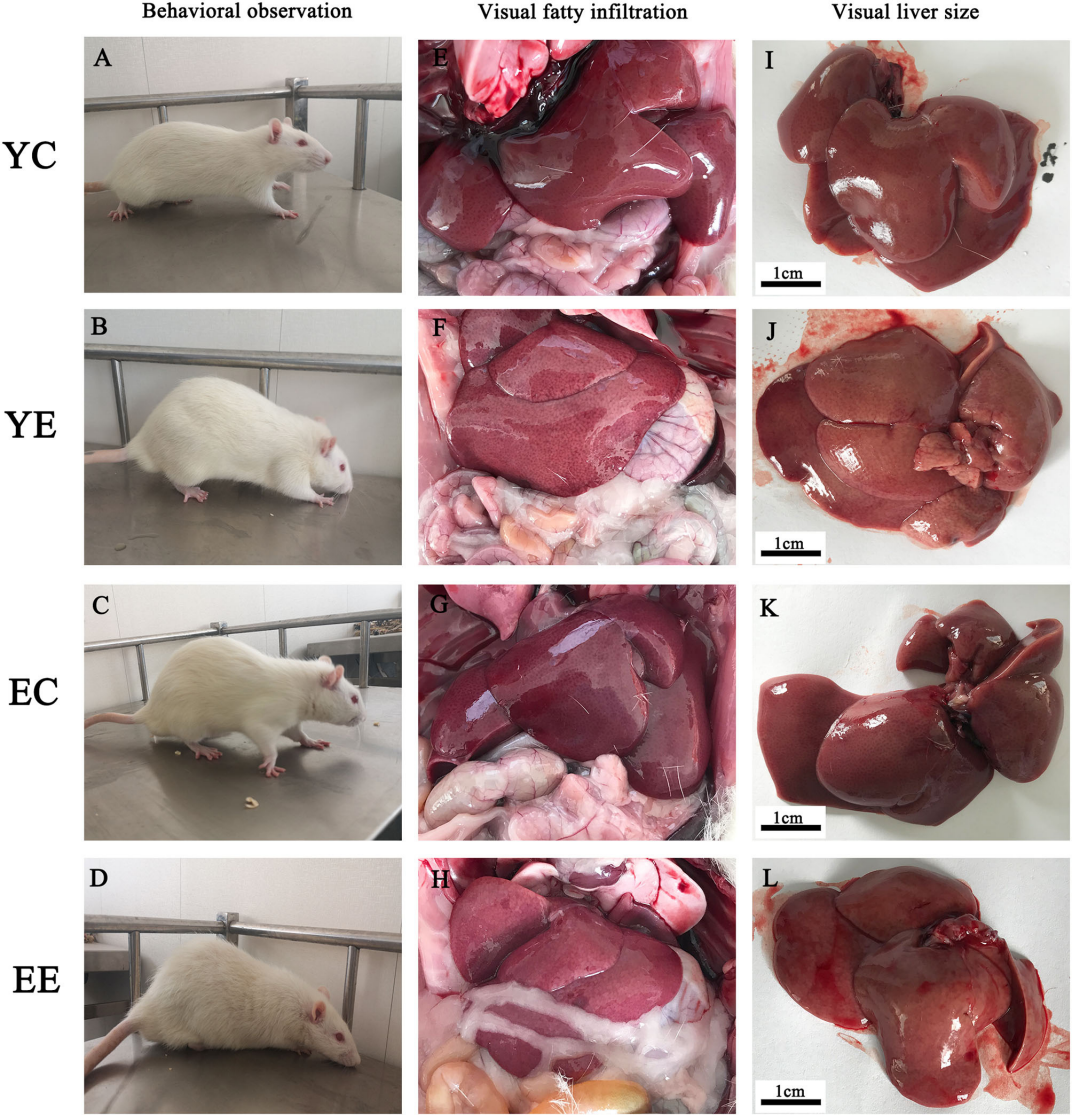
Effect of HFD on behavior and liver of rats. Comparisons among YC, YE, EC, and EE rats in appearance **(A–D)**, liver *in vivo* **(E–H)**, and liver *in vitro* **(I–L)**.

**Figure 3:**
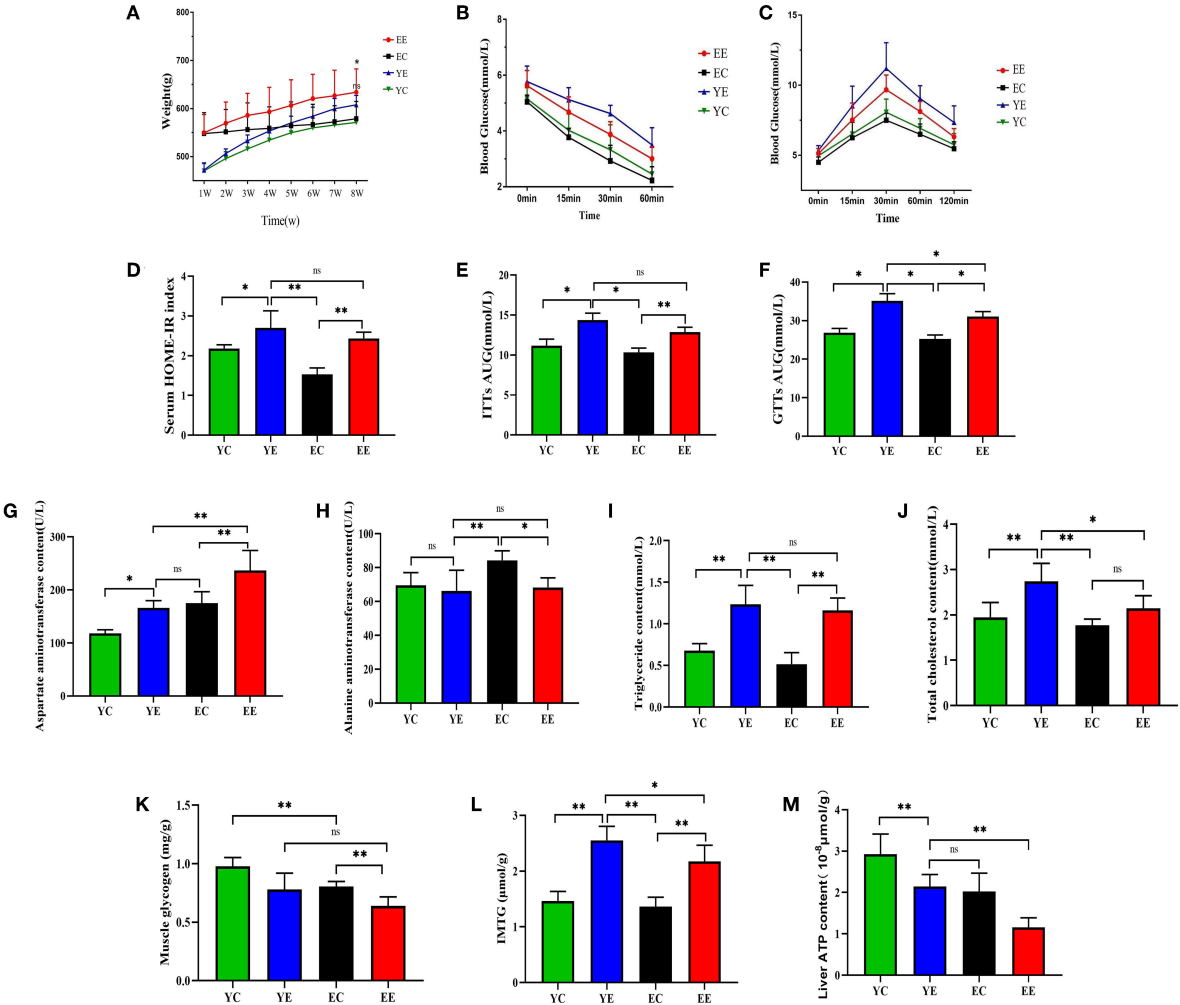
Effect of HFD on weight, insulin sensitivity, and blood biochemistry in EE, EC, YE, and YC rats. **(A)** The change in body weight every week. **(B)** ITTs. **(C)** GTTs. **(D)** HOMA-IR. **(E)** ITTs-AUC. **(F)** GTTs-AUC. **(G)** Effects of HFD by intervention on the plasma AST, **(H)** ALT, **(I)** TC, and **(J)** CHOL in rats. **(K)** Muscle glycogen. **(L)** IMTG. **(M)** Liver ATP content. *p < 0.05, **p < 0.01, ^ns^*p* > 0.05.

During the feeding process of 8 weeks, the weight of rats in YE and EE groups increased significantly, and the growth rate of YE group was the fastest, with a weight increase of about 32% (Table 2 and Figure 3A). In addition, in terms of appearance, the rats in YE and EE groups fed HFD have rough fur, yellow hair color, depressed spirit, unwilling to exercise, and like to lie down (Figures 2A–D).

**Table 2:** Changes in body weight (g) of rats in each group (*n* = 10, ± s).

**Group** **Time (week)**	**YC**	**YE**	**EC**	**EE**
1W	470.8 ± 14.8	472.0 ± 15.0	548.0 ± 43.3	549.8 ± 37.9
2W	496 ± 14.3	506.5 ± 9.6	551.5 ± 46.6	569.5 ± 44.0
3W	516 ± 18.2	533.0 ± 12.0	556.3 ± 55.7	585.8 ± 45.9
4W	534.3 ± 15.2	553.3 ± 9.9	558.8 ± 44.8	593.3 ± 51.1
5W	549.8 ± 11	570.3 ± 14.1	564.3 ± 49.7	606.5 ± 53.2
6W	559.8 ± 10.5	584.0 ± 18.9	566.8 ± 42.0	618.3 ± 50.4
7W	565.8 ± 7.9	597.0 ± 24.5	572.5 ± 33.3	626.5 ± 53.1
8W	571.3 ± 8.2	607.8 ± 19.4	578.8 ± 36.0	634.0 ± 48.5

*YC, young control group; YE, young experimental group; EC, elderly control group; EE, elderly experimental group*.

#### HFD Feeding Induces Significant Dyslipidemia, Insulin Resistance, and Hepatic Injury

When compared with YE group, the content of AST was significantly increased in EE (*P* < 0.01) (Figure 3G), but there was no significant change in ALT (*P* > 0.05) (Figure 3H). Serum triglyceride content was not significantly changed between the YE and EE groups (*P* > 0.05) (Figure 3I). The content of total cholesterol content (TC) in YE group was significantly higher than that in EE group (*P* < 0.05) (Figure 3J).

Intraperitoneal glucose tolerance test (IGTT) showed that compared with the YC and EC groups, the YE and EE groups developed severe glucose intolerance (Figure 3C) and the area under the blood glucose concentration curve was significantly increased (*P* < 0.05) (Figure 3F). Insulin tolerance test (ITT) results showed that compared with the YC group and the EC group, the YE group and the EE group were less sensitive to exogenous insulin (Figure 3B) and the area under the blood glucose concentration curve was significantly different (Figure 3E).

Based on these measurements, we calculated the insulin resistance index using the homeostatic model assessment (HOMA) index (Figure 3D and Table 3). The results showed that compared with the YC and EC groups, the HOMA-IR index of the YE and EE groups increased significantly, and there was no significant difference between the YE and EE groups (*p* > 0.05).

**Table 3:** Descriptive statistics of serum concentrations of FINs and FBG, and HOMA-IR in 40 rats.

**Group**	**FINs (μIU/mL)**	**FBG (mmol/L)**	**HOMA-IR**
YC	9.865 ± 0.382	4.975 ± 0.206	2.181 ± 0.094
YE	11.736 ± 1.741	5.175 ± 0.171	2.699 ± 0.426
EC	7.678 ± 0.768	4.500 ± 0.392	1.536 ± 0.161
EE	11.241 ± 1.218	4.875 ± 0.250	2.436 ± 0.157

*YC, young control group; YE, young experimental group; EC, elderly control group; EE, elderly experimental group; FINs, fasting serum insulin content; FBG, fasting blood glucose concentration*.

According to these results, it is shown that the 8-week HFD diet successfully induced insulin resistance in the rats of the YE and EE groups.

### Liver

**Figure 4:**
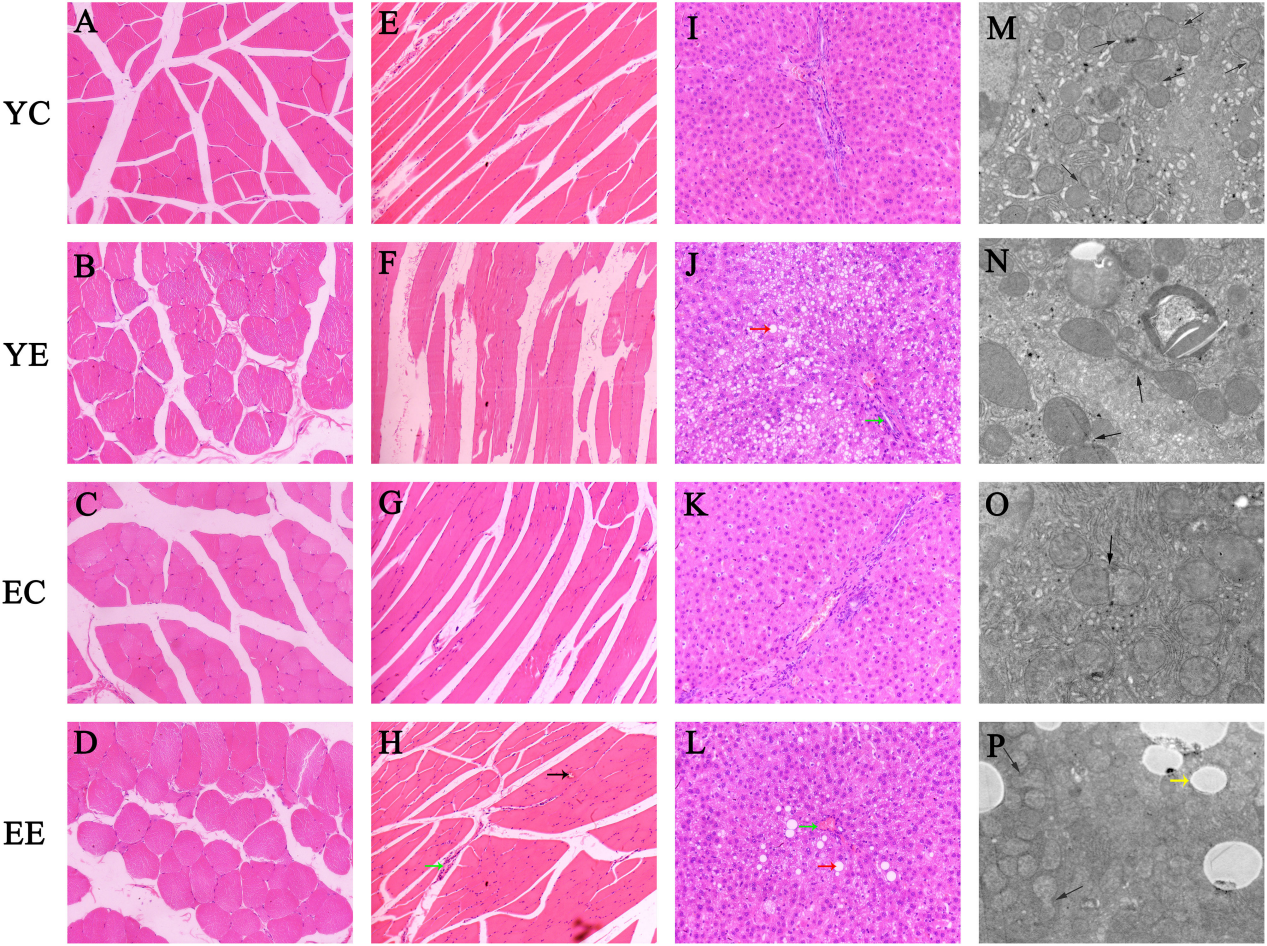
Hematoxylin–eosin staining of transverse **(A–D)** and longitudinal **(E–H)** sections of skeletal muscle. **(I–L)** Hematoxylin–eosin staining of liver tissue. (Green arrow: inflammatory cell infiltration; red arrow: vacuolar degeneration; black arrow: bleeding point). Original magnification: × 200. **(M–P)** Hepatic mitochondrial changes in each group's rats. Bar = 5.0um. (Yellow arrow: white fat droplet). Mitochondrial fission and fusion were evaluated with an inverted fluorescence microscope (Eclipse Ts2, Nikon, China).

**Figure 5:**
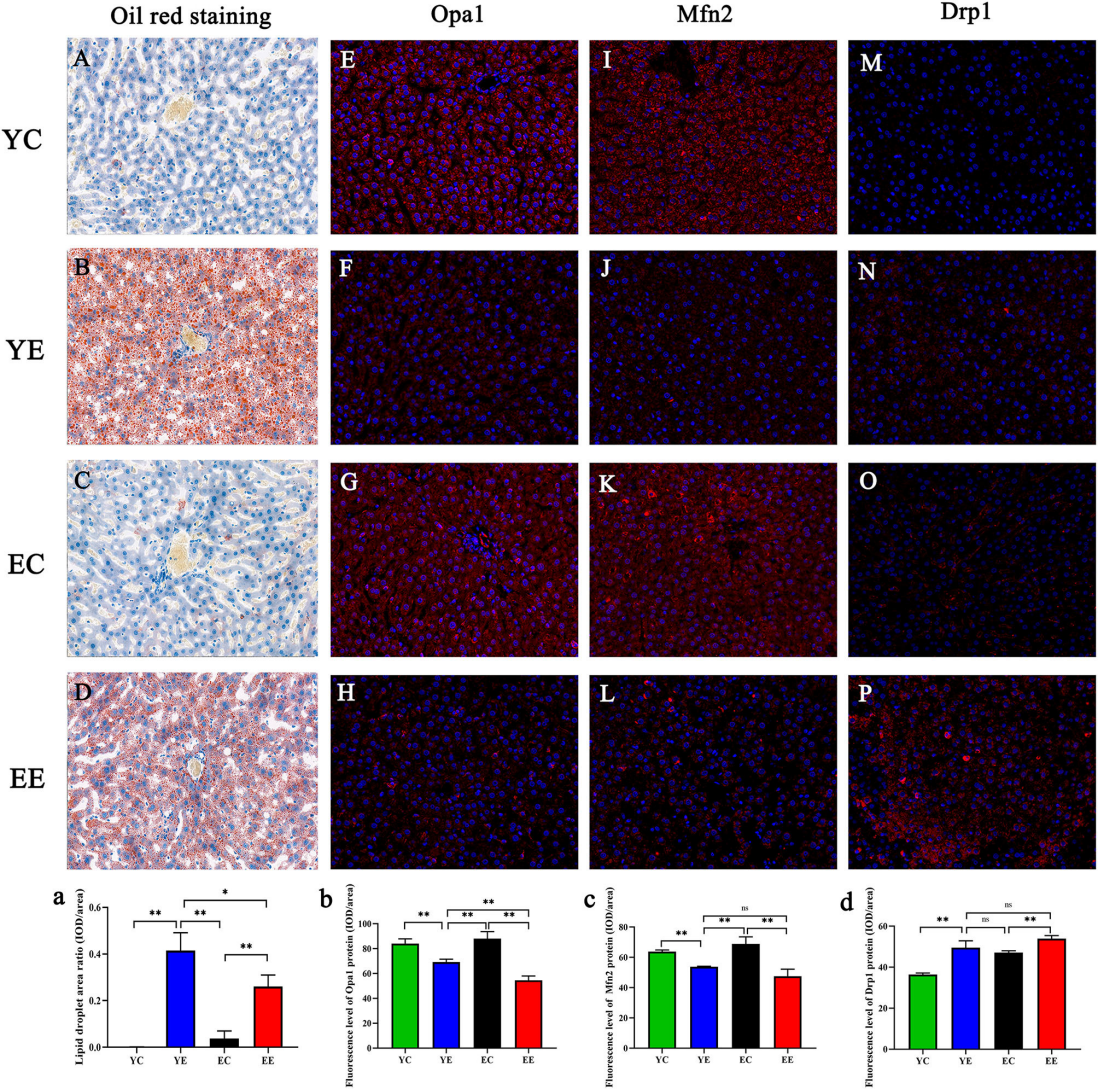
**(A–D)** Oil Red O staining of the liver. Original magnification: × 200. Opa1 **(E–H)**, Mfn2 **(I–L)**, and Drp1 **(M–P)** in liver mitochondria were labeled with red fluorescence in the cytoplasm and blue fluorescence in the nucleus. Effect of diet on hepatocytes of mice in each group. (a) Data analysis of liver lipid droplet area observed by Oil Red O staining. Opa1(b), Mfn2 (c), and Drp1 (d) protein fluorescence assay. *p < 0.05, **p < 0.01, ^ns^*p* > 0.05.

The rats in YE (Figure 2J) and EE (Figure 2L) groups with HFD-fed developed larger livers that were significantly heavier than those of the YC (Figure 2I) and EC (Figure 2K) group rats. Visually, the livers from the HFD-fed animals were distinguished from those of the control groups rats as yellowing in color, especially YE (Figure 2J). Compared with YC (Figure 2E) and EC (Figure 2G) groups, The presence of more fat around the liver can be observed in rats of the YE group (Figure 2F) and EE group (Figure 2H). Microscopic examination of tissue slides revealed higher hepatocyte lipid droplet accumulation in the livers from the YE and EE, compared to those from the YC and EC groups (Figures 5A–D). *Post-hoc* analysis showed that the degree of liver lipid droplet infiltration in YE group was higher than that in EE group (*P* < 0.05) (Figure 5A). TEM showed that there were relatively more liver mitochondria in YE group and EE group than in the control group, and the YE group had more mitochondria in fission state (Figures 4N,P).

### Skeletal Muscle

The H&E staining results of skeletal muscle in rats are shown in Figures 4A–D (Crosscutting) and Figures 4E–H (Slitting). In the YC (Figures 4A,E) and EC groups (Figures 4C,G), the skeletal muscle structure was intact, the muscle fibers were neatly arranged, and there were no fractures, increase in size or migration of nuclei, or inflammatory cell infiltration between muscle fibers. In the YE and EE groups, the muscle fibers were disordered and broken, and displayed inflammatory cell infiltration, non-uniform nuclei, and abnormal locations among the local muscle fibers. YE group was more severe than EE group.

The content of muscle glycogen in YE group was significantly lower than that in YC group (*P* < 0.05) and that in EE group was significantly lower than that in EC group (*P* < 0.01). There was no significant difference between YE group and EE group (*P* > 0.05) (Figure 3K). The content of IMTG in the experimental group was higher than that in the corresponding control group. There was a significant difference between YE group and EE group (*P* < 0.05), and the content of IMTG in YE group was the highest (Figure 3L).

### HFD Diet Promotes Mitochondrial Fission in Liver and Skeletal Muscle of Young Rats

We sought to identify the effect by hypercaloric diet exposure on rats' liver and skeletal muscle mitochondrial dynamics evidenced by changes in the expression of proteins involved in mitochondrial fusion (Mfn 2, Opa 1) and/ or fission (Drp 1).

**Figure 6:**
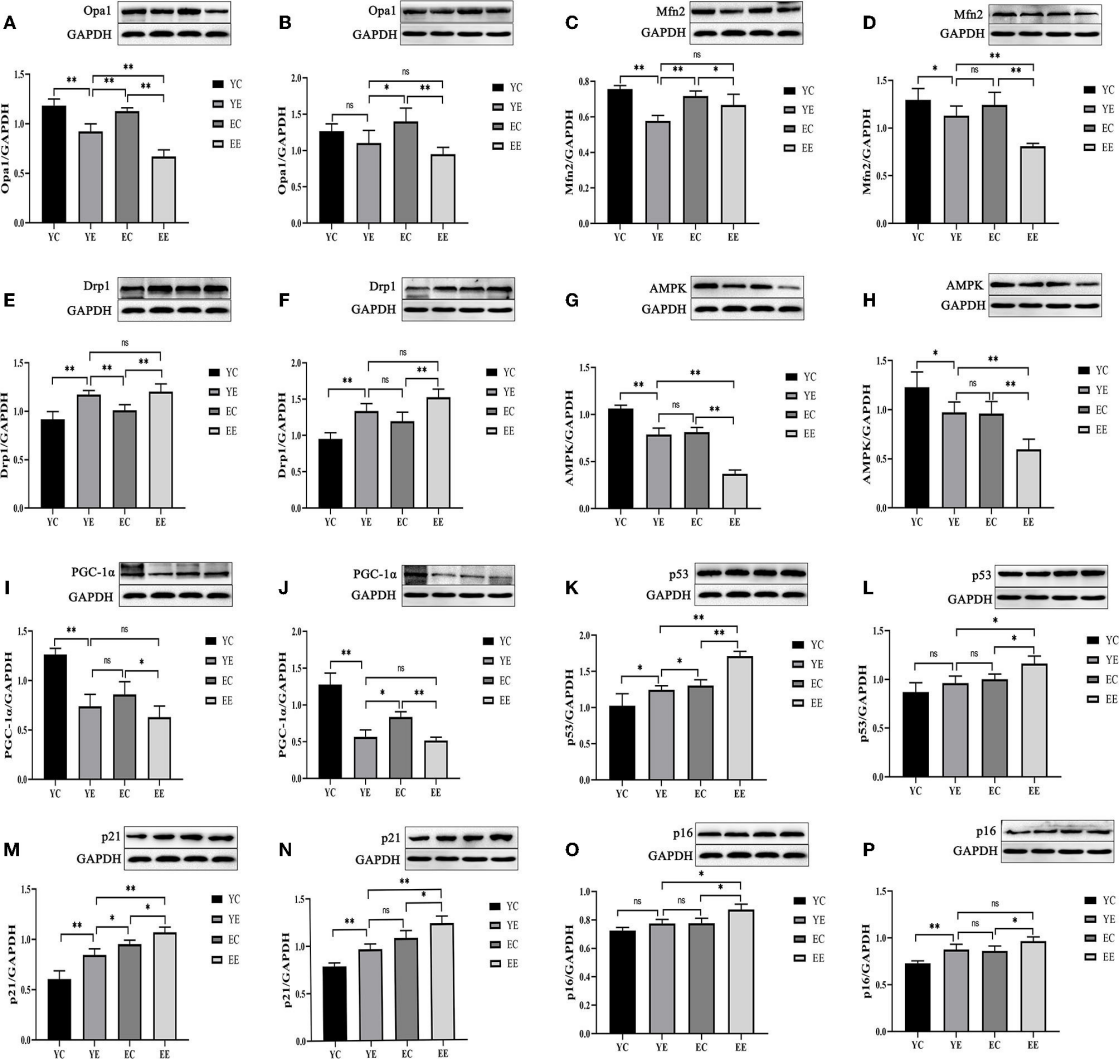
High-fat diet-induced mitochondrial fusion and division, energy stress, and aging in diabetic rats. Protein level of Opa1**(A,B)**, Mfn2 **(C,D)**, Drp1 **(E,F)**, AMPK **(G,H)**, PGC-1α **(I,J)**, p53**(K,L)**, p21 **(M,N)**, and p16 **(O,P)**. (Liver: **A,C,E,G,I,K,M,O**; Skeletal Muscle: **B,D,F,H,J,L,N,P**). *p < 0.05, **p < 0.01, ^ns^*p* > 0.05.

We found that Opa 1 protein in liver of YE group was significantly higher than that in EE group (*P* < 0.01) (Figure 6A), and there was no significant difference in skeletal muscle (*P* > 0.05) (Figure 6B). Mfn2 protein had no significant difference between YE group and EE group in liver (*P* > 0.05) (Figure 6C), but the difference is significant in skeletal muscle (*P* < 0.01) (Figure 6D). There was no significant difference in the expression of Drp 1 protein between the YE and EE groups in liver and skeletal muscle (*P* > 0.05) (Figures 6E,F).

These data suggest that hypercaloric diet exposure promoted mitochondrial fission in rats, especially young rats. Next, based on the results of protein expression found in the groups of YC, YE, EC, and EE, we quantified their immunofluorescence of Mfn 2, Opa 1, and Drp 1 genes in liver. We identified that the expression of Opa 1 in YE group was higher than that in EE group (*P* < 0.01) (Figures 5E–H and 5b), and there was no significant difference in the expression of Mfn2 and Drp1 between YE group and EE group (*P* > 0.05) (Figures 5C,D).

### High-Fat Diet Hinders Mitochondrial Biogenesis

**Figure 7:**
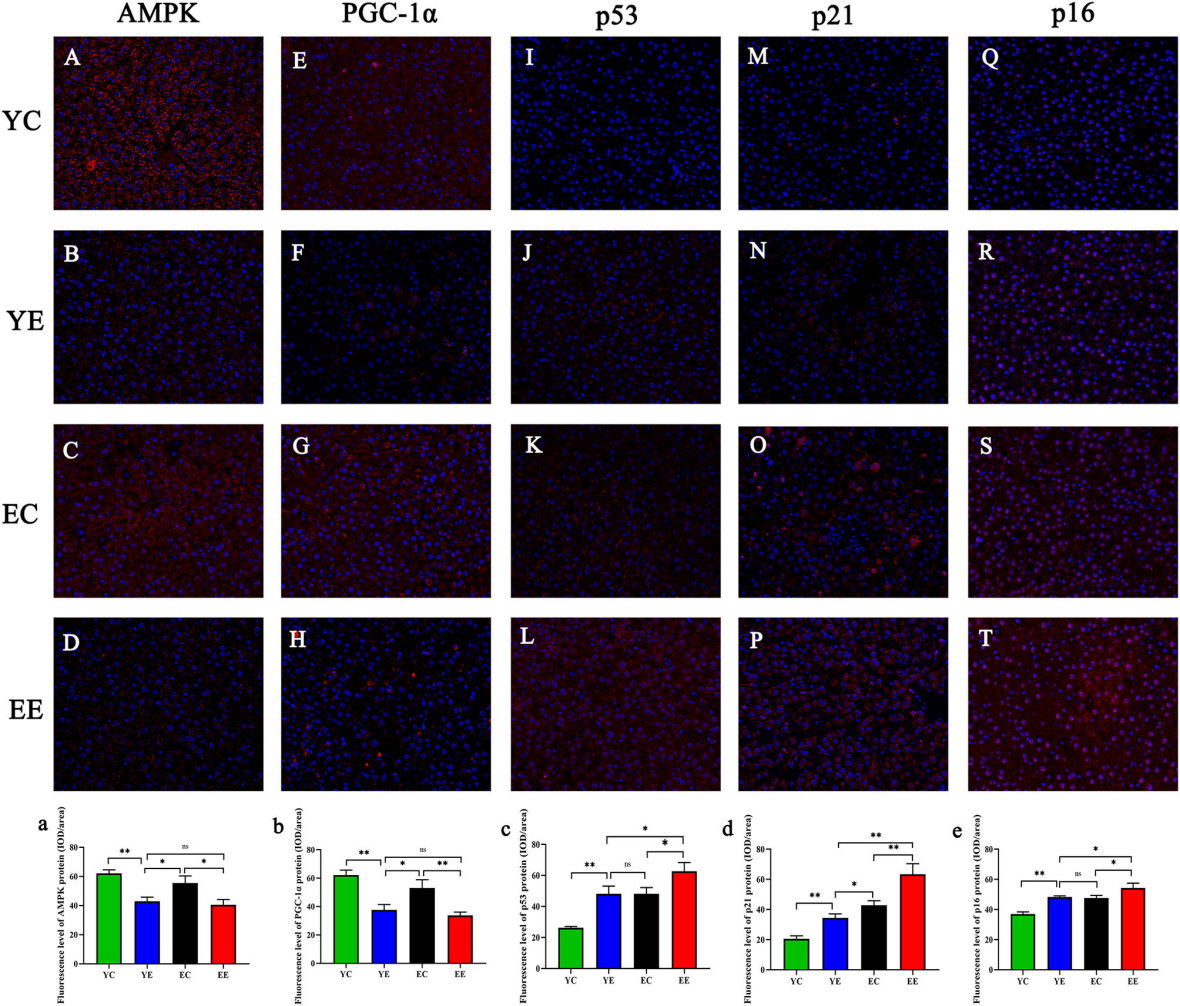
AMPK **(A–D)**, PGC-1α **(E–H)**, p53 **(I–L)**, p21 **(M–P)**, and p16 **(Q–T)** in liver mitochondria were observed via fluorescence microscopy. Magnification:200 ×. AMPK (a), PGC-1α (b), p53 (c), p21 (d), and p16 (e) proteins fluorescence assay. *p < 0.05, **p < 0.01, ^ns^*p* > 0.05.

We measured two proteins representative of mitochondrial biosynthesis and energy metabolism, AMPK (Figures 7A–D and 7a) and PGC-1α (Figures 7E–H and 7b), in liver and skeletal muscle, as well as ATP content in liver (Figure 3M).

The results show, in liver and skeletal muscle, that the expression of AMPK protein in YE group was significantly higher than that in EE group (*P* < 0.01), but there was no significant change in YE group and EC group (*P* > 0.05) (Figures 6G,H); the expression of PGC-1α protein in liver (Figure 6I) and skeletal muscle (Figure 6J) of YE group was not significantly different from that of EE group (*P* > 0.05). In addition, the results of detecting the ATP content in the liver showed that the YE group was significantly higher than the EE group (*P* < 0.01) (Figure 3M).

### Senescence in Hepatic and Skeletal Muscle Tissue

Hepatic and skeletal muscle senescence was investigated via Western blot by measuring the protein expression of p53, p21, and p16, which are considered senescence markers.

After 8 weeks of HFD feeding, in the liver, the protein expressions of p53 and p21 in the EE group were significantly higher than those in the YE group (*P* < 0.01) (Figures 6K,M), and the p16 protein of EE group was also significantly upregulated compared with YE group (*P* < 0.05) (Figure 6O). In the skeletal muscle, the expression of p53 and p21 proteins of EE was significantly upregulated compared with the YE group (*P* < 0.05 and *P* < 0.01) (Figures 6L,N), but there was no significant difference in the expression of p16 protein in two groups (*P* > 0.05) (Figure 6P).

Next, we performed immunofluorescence quantitative analysis of p53 (Figures 7I–L and 7c), p21 (Figures 7M–P), and p16 (Figures 7Q–T and 7e) in liver. There was a significant difference in p53 protein expression between YE and EE groups (*P* < 0.05), but no significant difference between YE and EC groups (*P* > 0.05) (Figure 7C). There was a significant increase in p21 protein expression in the EE group compared to the YE group (*P* < 0.01) and in the EC group (*P* < 0.05) (Figure 7D). There was also a significant difference in p16 protein expression between the YE and EE groups (*P* < 0.05), but no significant difference between the YE and EC groups (*P* > 0.05) (Figure 7E).

Overall, the EE group had the most severe aging degree, but the YE group had almost the same degree of aging as the EC group. It is clear from the above results that the high-energy diet induces insulin resistance in rats, which also simultaneously promotes aging of the organism.

## Discussion

Insulin resistance is the common basis of a variety of metabolic diseases, such as obesity, T2DM, and cardiovascular diseases (22–25). Insulin resistance caused by excess energy mostly occurs in elderly animals. However, more and more evidence shows that the incidence rate of diabetes and insulin resistance is also increasing in young individuals (26, 27). Therefore, insulin resistance in young individuals also needs to be paid attention. *In vivo*, insulin resistance not only disturbs the metabolic process of sugar and fat, but also affects the productivity function of the body, especially the energy biogenesis ability of mitochondria (28, 29). At the same time, insulin resistance will also lead to a large amount of fat deposition in the liver and skeletal muscle and may even aggravate the process of aging (30). Young individuals have stronger cellular metabolic capacity and stronger mitochondrial compensation than older individuals (31). But since insulin resistance and diabetes have occurred, whether similar changes have taken place with older individuals, whether mitochondrial function of young individuals has been severely affected, and whether there has been significant aging in cells has not been reported. Therefore, this study focuses on the changes in mitochondrial function and the degree of cell aging in young insulin-resistant individuals.

To study the effect of HFD-induced insulin resistance on mitochondrial function of liver and skeletal muscle in young and old rats, we used a kind of commercialized high-fat diet to construct the insulin resistance model, which is a representative model of diabetes with related metabolic complications in young and old body (32–34). Clinically, glucose tolerance test, insulin tolerance test, and HOMA-IR are often used to detect insulin resistance (21, 35). Because when insulin resistance occurs, the sensitivity of cells to insulin decreases significantly, which resulted in the elevation of the body's fasting plasma glucose level ultimately (36). This phenomenon was also found in our experiment, even though the blood glucose concentration decreased after prolonged starvation, and the blood glucose in the YE and EC groups was still higher than that in the YC and EC groups. In the glucose tolerance test and insulin tolerance test, it was also found that the blood glucose concentration of the experimental group was higher than that of the control group. In our experiment, the fasting blood glucose concentration and fasting insulin content of rats in each group were incorporated into the HOMA-IR formula to calculate the insulin resistance index. The results showed that the insulin resistance index of the experimental group was significantly higher than that of the control group, indicating that a significant insulin resistance phenomenon occurred after feeding a high-energy diet, which shows that the insulin resistance rat model established in this experiment was successful. In addition, we also found that the YE group had the highest HOMA-IR index, which would normally define the most severe insulin resistance in the YE group, but this is not true, because the HOMA-IR formula is based on multiplying the fasting blood glucose concentration and the serum insulin concentration. However, with the senility of the body tissue in the elderly, the metabolism of pancreatic cells decreases, which causes the gradual aging of the pancreas, which in turn affects the secretion of insulin. Therefore, compared with young rats, the serum insulin concentration of aged rats must be lower, so the HOMA-IR formula cannot fully reflect the degree of insulin resistance in young and old rats. Therefore, this experiment combines mitochondrial dynamics and histopathology to analyze various aspects, and the focus was on differences in liver and skeletal muscle impaired from high-fat diets in young rats compared to older healthy rats.

When carbohydrates and other nutrients are ingested into the body, the body will timely convert them into energy available for cells through various metabolic reactions. When too much energy is continuously ingested, this energy will be transported to liver, skeletal muscle, and other tissues in time for storage (37, 38). In the body, energy storage and utilization will maintain a relative balance and have a certain upper limit of regulation. When the rats in the experimental group ingest too much energy, the balance between energy storage and energy utilization in the body is broken, even exceeding the upper limit of the body. The phenomenon of the high insulin resistance index in the YE and EE groups found in this experiment should also be the result that the continuous intake of energy leads to the premature reaching of the upper limit of energy storage and utilization. This result may also be related to the relatively small intake of food in the elderly rats. On the contrary, it also shows that even young individuals will have significant insulin resistance after eating a large amount of high-energy feed, which is not what we usually think of as “young individuals have strong energy processing ability.” Young individuals also have an upper limit on energy utilization and storage. If they exceed this upper limit, they will lead to insulin resistance. When animals become obese and insulin-resistant, blood glucose becomes more difficult to be controlled due to the combination of excess hepatic glucagon action and multi-tissue insulin resistance (39, 40).

Studies of liver function in diabetic organisms showed that 28.0 to 36.8% of patients had liver dysfunction (41, 42). Prolonged fatty infiltration of the liver in the diabetic has been shown to lead to hepatic cirrhosis (43). As a result, liver injury is more severe in young rats than in older rats who eat the same high-calorie diet. We thought that this is related to the strong metabolic ability of the rats in the YE group, which can deliver excess energy to the liver for storage timely. In this experiment, compared with other groups of rats, the weight of the rats in the YE group was the heaviest, and the results of Oil Red O staining also indicated that the liver lipid droplet infiltration in the YE group was significantly more extensive than that in the EE group. Meanwhile, it can be seen from the results of ALT and AST that the degree of liver injury in YE group is significantly lower than that in EE group, which explained the strong self-metabolism and repairability of young rats, which can repair or remove damaged hepatocytes in time. In addition, due to the more aging state of EE group, the self-repairability and clearing ability of body tissues are relatively weak.

Mitochondria are always in the process of dynamic regulation including fission and fusion to meet the different energy needs of cells and then maintain mitochondrial and cellular functions (44, 45). In obesity and type 2 diabetes, reduced mitochondrial contents have been reported (44, 46). Under a transmission electron microscope, the mitochondria in the livers of experimental and control rats were observed, and the fusion process of liver mitochondria was more pronounced in the control group fed with normal feed, while in the experimental group fed with high-energy feed, it was the division process that was more pronounced. Mitochondrial fusion is promoted by mitofusin 1 (Mfn1), mitofusin 2 (Mfn2), and optic atrophy 1 (Opa 1), while fission is controlled by proteins such as Drp1 (44, 47). It was found that the expression of two kinds of fusion protein (Mfn 2 and Opa 1) in the control group was significantly higher than that in the experimental group. This is because the rats in the control group were only fed with ordinary feed, which would have a greater demand for energy. Therefore, the mitochondria in the liver of the rats in the control group were fused to generate more energy for cell utilization. There are more fusion phenomena in liver mitochondria of young control group rats, which may be related to the fact that young control group rats are in the youth stage and their own metabolism and functions are relatively vigorous, so they will produce more ATP through mitochondria for cell utilization. In addition, endoplasmic reticulum stress can inhibit mitochondrial fusion caused by fusion proteins Mfn 1 and Mfn 2 and upregulate mitochondrial division proteins to increase mitochondrial division. Drp 1 is an important protein that regulates mitochondrial fission and clears damaged parts of the cell by regulating autophagy (48). By detecting the expression of Drp 1, it was found that the expression of division protein in the experimental group was also higher than that in the control group. This indicates that continuous high-energy diet will reduce the body's demand for energy. Therefore, mitochondrial division will occur in the liver tissue of rats in the experimental group, which is also the repair of mitochondria after injury. There was no significant difference in the expression of Drp 1 protein between the YE and EE groups, which indicated that the liver and skeletal muscle of the two groups suffered the same degree of injury. However, due to the stronger metabolism and reparability of the young body, the damaged components can be treated faster than the old body. Therefore, according to the results, it can be known that the reparability of the liver and skeletal muscle of the YE group is stronger than that of the EE group.

The study found that skeletal muscle mainly provides energy by oxidizing FFA at rest (49, 50). When high-fat diet and obesity will cause the increase in fatty acids in blood exceeding the oxidation capacity of skeletal muscle, excessive esterified fatty acids are deposited in skeletal muscle, which will affect insulin-mediated signal transduction and lead to insulin resistance (51). The results of this experiment showed that the level of IMTG in the YE group was significantly lower than that in the EE group. This may be because the skeletal muscle of young rats still has a strong oxidative capacity and a relatively high therapeutic level of fatty acid deposition compared to old rats. However, the IMTG levels in the YE group were higher than those in the EC group, suggesting that long-term consumption of a high-fat diet in young people would impair the oxidative capacity of skeletal muscle to fatty acids, even lower than the oxidative level of healthy older bodies, resulting in muscle “premature aging.” At the same time, muscle mitochondrial volume was reduced in subjects with insulin resistance or type 2 diabetes (52), and this confirms that the muscle fatty acid oxidation ability of YE rats is stronger than that of EE group. Given that the ability to store glucose in the form of glycogen is a hallmark of insulin sensitivity (53), we measured glycogen content in skeletal muscle. Compared with YC group, the content of muscle glycogen in EC group decreased significantly, indicating that the content of muscle glycogen decreased with the increase in age. This may be because the skeletal muscle in aged rats has an impaired ability to upregulate glycogen synthesis and the insulin function of the elderly body is more defective than that of the young body. But there was no significant difference in muscle glycogen content between YE group and EE group, and it shows that the decrease in insulin sensitivity of skeletal muscle in two groups is similar, which all leads to the decrease in glucose absorption by skeletal muscle. This maybe because the impairment occurs early in intracellular glucose metabolism concomitantly with an initial, rapid, and disproportionate increase in fat mass, and compared with the old rats, the young rats have stronger glycogen synthesis ability (54).

There is growing evidence support that mitochondrial dysfunction links to diabetes (55), while it has been known for a long time that AMPK and PGC-1α act as two major regulators of mitochondrial function (56). AMPK is activated in response to an increase in the cellular AMP/ATP ratio, indicative of an energy deficit, and acts to switch the cellular metabolic program from ATP consumption to ATP production (57). We therefore measured AMPK and PGC-1α expression in the liver and skeletal muscle of the rats, as well as liver ATP content. The results showed that the protein expression of AMPK and PGC-1α was lower in the YE and EE groups and higher in the YC and EC groups. In addition, the ATP content was lower in the YE and EE groups compared to the YC and EC groups. It indicates that rats in the experimental groups fed high-energy diets have lower energy requirements; at this time, the reserve capacity of ATP is reduced, the expression of AMPK and PGC-1α is downregulated, and the biosynthesis of mitochondria is reduced. Rats in the control group fed a normal diet would not have excess energy, have a higher ATP reserve capacity, and increase mitochondrial biosynthesis by regulating the activation of AMPK and PGC-1a to produce more ATP for cellular use. AMPK protein expression in the YE group was significantly higher than that in the EE group. This suggests that although both the EE and YE groups were fed high-fat diets and both developed insulin resistance, the rats of the YE group may have a greater energy requirement. Therefore, young rats may increase mitochondrial biosynthesis by activating AMPK and PGC-1α. In conclusion, compared with old rats, youth have a high metabolic ability and stronger energy utilization and storage capacity, and when the body requires energy, it immediately mobilizes energy-regulating factors to meet the body's energy needs. This tight regulation of energy also serves as a protection for the body itself against continuous high-energy diet-induced metabolic diseases, such as diabetes. However, due to poor metabolism and self-regulation, aged rats are unable to use or store excessive energy in a timely manner, thus greatly increasing the risk of developing metabolic diseases such as diabetes mellitus.

Numerous studies have shown diabetes affects endothelial cell fate by increasing the expression of p53, p21, and p16 (58). p21 belongs to the cyclin-dependent kinase (CDK) inhibitors that, in concert with various tumor suppressor proteins, such as p53 and p16, induce inhibition of DNA replication and control anti-proliferative programs (59, 60). However, p21 together with the tumor suppressor proteins p53 and p16 is not only an important mediator of quiescence-like growth arrest but also of senescence (61, 62). Remarkably, conditional overexpression of p21 has been reported to be associated with growth arrest and phenotypic features of senescence (63). In this experiment, compared with YC group, the expressions of aging proteins p53, p21, and p16 in liver and skeletal muscle increased in YE group. It shows that the insulin resistance model of rats induced by high-energy feed will increase the aging degree of young rats. Compared with the YE group, the aging phenotype and the expression of aging protein in the EE group were significant increase. During aging, ATM or ATR kinase will activate p53, which inhibits the activity of CDK and blocks the process of cell cycle by upregulating the downstream target gene p21. In addition, p16 can also increase its expression caused by the production of ROS and mitochondrial dysfunction, arrest the cell cycle through p16-pRb pathway, and promote cell aging. Some studies have shown that cell senescence is often beneficial in clinic. When cells accumulate a lot of injury, they will rely on cell cycle points and stress relief mechanism to maintain the stability of cell cycle. However, with the gradual accumulation of injury, the stability of cells will become worse and worse, which will start aging, apoptosis, and other procedures to prevent the malignant development of cells (64). Therefore, the aging of rats in the old experimental group and the young experimental group is more serious. The EE group showed the most severe aging according to the expression levels of p53, p21, and p16 proteins. From the results, the high-fat diet caused the most severe injury to the EE rats. Compared with the YC group, the degree of aging was significantly worse in the YE group, suggesting that a high-fat diet-induced diabetes in young leads to premature aging of the body. Notably, it is possible that the degree of injury was similar in the YE and EE groups, but because the young can clear the broken components of the cells to divide faster and repair the injury better, it resulted in a slightly lower degree of senescence in the YE group compared with the EC.

In summary, our results show that young rats fed a high-fat, high-energy diet have decreased mitochondrial biogenesis, mitochondrial injury, and cellular aging in liver and skeletal muscle close to those of older rats fed an ordinary diet. This suggests that even if young people are stronger than older people, if they suffer from diabetes due to chronic feeding of high-fat, high-energy foods, they will have more severe mitochondrial dysfunction than healthy older people and will cause premature aging of young body cells.

## Data Availability Statement

The original contributions presented in the study are included in the article/supplementary materials, further inquiries can be directed to the corresponding author/s.

## Ethics Statement

The animal study was reviewed and approved by the Tab of Laboratory Animal Welfare and Ethics Committee of Northeast Agricultural University.

## Author Contributions

JWa and JWu conceptualized the study. JWa and WL conducted the formal analysis. JWa wrote the original draft. XW, RL, TL, and JX wrote, reviewed, and edited the article. All authors have read and agreed to the published version of the manuscript.

## Conflict of Interest

The authors declare that the research was conducted in the absence of any commercial or financial relationships that could be construed as a potential conflict of interest.

## Publisher's Note

All claims expressed in this article are solely those of the authors and do not necessarily represent those of their affiliated organizations, or those of the publisher, the editors and the reviewers. Any product that may be evaluated in this article, or claim that may be made by its manufacturer, is not guaranteed or endorsed by the publisher.

## References

[1] Qiang G Li Z Yang X Qi X Shi L Zhang H. Effect of valsartan on the pathological progression of hepatic fibrosis in rats with type 2 diabetes. Eur J Pharmacol. (2012) 685:156–64. 10.1016/j.ejphar.2012.04.028 22546234

[2] Biobaku F Ghanim H Batra M Dandona P. Macronutrient-mediated inflammation and oxidative stress: relevance to insulin resistance, obesity, and atherogenesis. J Clin Endocrinol Metabol. (2019). 10.1210/jc.2018-01833 31219543

[3] Barazzoni R Cappellari GG Ragni M Nisoli E. Insulin resistance in obesity: an overview of fundamental alterations. Eat Weight Disord. (2018) 23:149–57. 10.1007/s40519-018-0481-6 29397563

[4] Chang YH Chang DM Lin KC Shin SJ Lee YJ. Visfatin in overweight/obesity, type 2 diabetes mellitus, insulin resistance, metabolic syndrome and cardiovascular diseases: a meta-analysis and systemic review. Diab Metabol Res Rev. (2011) 27:515–27. 10.1002/dmrr.1201 21484978

[5] Bekhite M González-Delgado A Hübner S Haxhikadrija P Kretzschmar T Müller T. The role of ceramide accumulation in human induced pluripotent stem cell-derived cardiomyocytes on mitochondrial oxidative stress and mitophagy. Free Radical Biol Med. (2021) 6:2016. 10.1016/j.freeradbiomed.2021.02.016 33705961

[6] Eweda SM Ali MA El-Bary H El-Sokkary NH Helmy MH Kamel AN. Bitter gourd extract improves glucose homeostasis and lipid profile via enhancing insulin signaling in the liver and skeletal muscles of diabetic rats. Asian Pac J Trop Biomed. (2021) 11:9. 10.4103/2221-1691.319569

[7] Bronczek GA Soares GM de Barros JF Vettorazzi JF Kurauti MA Marconato-Júnior E. Resistance exercise training improves glucose homeostasis by enhancing insulin secretion in c57bl/6 mice. Sci Rep. (2021) 11: 8574. 10.1038/s41598-021-88105-x 33883630 PMC8060292

[8] Liang Z Zou T Alberto GN Bo W Zhu MJ Min D. Raspberry alleviates obesity-induced inflammation and insulin resistance in skeletal muscle through activation of amp-activated protein kinase (ampk) α1. Nutr Diabetes. (2018) 8:39. 10.1038/s41387-018-0049-6 29961765 PMC6026595

[9] Fan L Cahill-Smith S Geng L Du J Brooks G Li JM. Aging-associated metabolic disorder induces nox2 activation and oxidative damage of endothelial function. Free Radic Biol Med. (2017) 108:940–51. 10.1016/j.freeradbiomed.2017.05.008 28499911 PMC5489050

[10] Soysa A Langaas M Jakic A Shojaee-Moradie F Mostad IL. The fat mass and obesity-associated (fto) gene allele rs9939609 and glucose tolerance, hepatic and total insulin sensitivity, in adults with obesity. PLoS ONE. (2021) 16:e0248247. 10.1371/journal.pone.0248247 33684170 PMC7939351

[11] β-cells in youth with impaired glucose tolerance or early type 2 diabetes secrete more insulin and are more responsive than in adults. Pediat Diab. (2020) 21:13113. 10.1111/pedi.13113 32902875 PMC7642023

[12] Catchpole B Kennedy LJ Davison LJ Ollier WE. Canine diabetes mellitus: from phenotype to genotype. J Small Ani Pract. (2008) 49:4–0. 10.1111/j.1748-5827.2007.00398.x 17617163

[13] Castorani V Polidori N Giannini C Blasetti A Chiarelli F. Insulin resistance and type 2 diabetes in children. Annals Pedia Endocrinol Metabol. (2020) 25:217–26. 10.6065/apem.2040090.045 33401880 PMC7788344

[14] Habeeb Mosa RM. Evaluation of Pharmacological Modification of Growth Hormone Secretagogue Receptor in a Mouse Model of Non-Obese Type 2 Diabetes Mellitus. Brisbane, QLD: The University of Queensland (2017).

[15] Huxley R James WPT Barzi F Patel JV Woodward M. Ethnic comparisons of the cross-sectional relationships between measures of body size with diabetes and hypertension. Obes Rev. (2010) 9 Suppl 1:53–61. 10.1111/j.1467-789X.2007.00439.x 18307700

[16] Owen KR. Treating young adults with type 2 diabetes or monogenic diabetes. Best Pract Res Clin Endocrinol Metab. (2016) 30:455–67. 10.1016/j.beem.2016.05.002 27432078

[17] Prevalence of type 1 and type 2 diabetes among children and adolescents from 2001 to 2009. Jama. (2014) 311:1778. 10.1001/jama.2014.3201 24794371 PMC4368900

[18] Walia R Singh A Aggarwal A Thapa B Guptasarma ML Bhansali A. Look beyond gluten in short stature with celiac disease – a prospective, interventional study. Indian J Pediat. (2020) 8:550–4. 10.1007/s12098-020-03543-1 33095395

[19] Wan W Jiang B Sun L. Metabolomics reveals that vine tea (*ampelopsis grossedentata*) prevents high-fat-diet-induced metabolism disorder by improving glucose homeostasis in rats. PLoS ONE. (2017) 12:e0182830. 10.1371/journal.pone.0182830 28813453 PMC5558946

[20] Dolo PR Huang K Widjaja J Li C Zhu X Yao L. Distal gastric mucosa ablation induces significant weight loss and improved glycemic control in type 2 diabetes sprague-dawley rat model. Surg Endos. (2019) 34:4336–46. 10.1007/s00464-019-07200-3 31630290

[21] Strage EM Ley CJ Forkman J Öhlund M Stadig S Bergh A. Homeostasis model assessment, serum insulin and their relation to body fat in cats. BMC Veterinary Res. (2021). 17:2729. 10.1186/s12917-020-02729-1 33461546 PMC7814573

[22] Huang Y Huang T Zhen X Li Y Cheng N. A selective sphingomyelin synthase 2 inhibitor ameliorates diet induced insulin resistance via the irs-1/akt/gsk-3β signaling pathway. Pharmazie. (2019) 74:553–8. 10.1691/ph.2019.9310 31484596

[23] Réggami Y Benkhaled A Boudjelal A Berredjem H Amamra A Benyettou H. Artemisia herba-alba aqueous extract alleviated oxidative stress and atherogenic dyslipidaemia in rats with fructose-induced metabolic syndrome. In: Séminaire international (SINAAN19): "Avancées sur les Antioxydants Naturels: Sources, mécanismes d'action et valorisation en santé. Algérie: Université de Bejaia (2019). p. 541–50.

[24] Baldeweg SE Golay A Natali A Balkau B Del PS Coppack SW. Insulin resistance, lipid and fatty acid concentrations in 867 healthy europeans. European group for the study of insulin resistance (egir). Eu J Clin Invest. (2015) 30:45–52. 10.1046/j.1365-2362.2000.00597.x 10620001

[25] Huang P Wang B Shu-Guo WU Chen JZ Medicine CC Hospital DC. Correlation among insulin resistance,insulin secretion,the severity of disease and prognosis in patients with severe sepsis. Hainan Med J. (2015).

[26] Nelson WR. Why is the rate of incidence of diabetes increasing? Italian General Rev Dermatol. (2015). 10.5281/zenodo.16337

[27] Lin CH Li HY. Hypertension is associated with a higher progression rate of insulin resistance and the incidence of diabetes. Diabetes. (2018) 67(Supplement 1):1510. 10.2337/db18-1510-P

[28] Nakamura S Takamura T Matsuzawa-Nagata N Takayama H Misu H Noda H. Palmitate induces insulin resistance in h4iiec3 hepatocytes through reactive oxygen species produced by mitochondria. J Biol Chem. (2009) 284:14809–18. 10.1074/jbc.M901488200 19332540 PMC2685662

[29] Coletta DK Mandarino LJ. Mitochondrial dysfunction and insulin resistance from the outside in: extracellular matrix, the cytoskeleton, and mitochondria. Am J Physiol Endocrinol Metab. (2011) 301. 10.1152/ajpendo.00363.2011 21862724 PMC3214002

[30] Patel SA Hoehn KL Lawrence RT Sawbridge L Talbot NA Tomsig JL. Overexpression of the adiponectin receptor adipor1 in rat skeletal muscle amplifies local insulin sensitivity. Endocrinology. (2012) 11:5231–46. 10.1210/en.2012-1368 22989629 PMC3498583

[31] Kosaki K Kamijo-Ikemori A Sugaya T Tanahashi K Kumagai H Sawano Y. Relationship between exercise capacity and urinary liver-type fatty acid-binding protein in middle-aged and older individuals. Clin Exp Nephrol. (2017) 2:1385. 10.1007/s10157-017-1385-x 28197733

[32] Tanaka S Hayashi T Toyoda T Hamada T Shimizu Y Hirata M. High-fat diet impairs the effects of a single bout of endurance exercise on glucose transport and insulin sensitivity in rat skeletal muscle. Metabol-Clin Exp. (2007) 56:1719–28. 10.1016/j.metabol.2007.07.017 17998027

[33] Gan KX Wang C Chen JH Zhu CJ Song GY. Mitofusin-2 ameliorates high-fat diet-induced insulin resistance in liver of rats. W J Gastroenterol Wjg. (2013) 1572–81. 10.3748/wjg.v19.i10.1572 23538485 PMC3602474

[34] Zong H Armoni M Harel C Karnieli E Pessin JE. Cytochrome p-450 cyp2e1 knockout mice are protected against high-fat diet-induced obesity and insulin resistance. Am J Physiol Endocrinol Metab. (2015) 302:E532. 10.1152/ajpendo.00258.2011 22185839 PMC3311288

[35] Ausk KJ Boyko EJ Ioannou GN. Insulin resistance predicts mortality in nondiabetic individuals in the u.s. Diab Care. (2010) 33:1179–85. 10.2337/dc09-2110 20200308 PMC2875420

[36] Ruhul-Kabir M Kamrul-Hasan AB Burhan-Uddin SA Majumder M Rahman H Nazmul-Islam AF. Serum magnesium status and its correlation with insulin resistance in newly diagnosed patients with type 2 diabetes mellitus. Sri Lanka J Diab Endocrinol Metabol. (2019) 1:7397. 10.4038/sjdem.v9i1.7367

[37] Aydin S Kuloglu T Aydin S Eren MN Celik A Yilmaz M. Cardiac, skeletal muscle and serum irisin responses to with or without water exercise in young and old male rats: cardiac muscle produces more irisin than skeletal muscle. Peptides. (2014) 52:68–73. 10.1016/j.peptides.2013.11.024 24345335

[38] Iossa S Mollica MP Lionetti L Crescenzo R Botta M Liverini G. Skeletal muscle oxidative capacity in rats fed high-fat diet. Int J Obes. (2002) 26:65–72. 10.1038/sj.ijo.0801844 11791148

[39] Skeletal muscle salt inducible kinase 1 promotes insulin resistance in obesity. Mol Metabol. (2016) 5:34–46. 10.1016/j.molmet.2015.10.004 26844205 PMC4703802

[40] Djohan YF Badia E Bonafos B Fouret G Lauret C Dupuy AM. High dietary intake of palm oils compromises glucose tolerance whereas high dietary intake of olive oil compromises liver lipid metabolism and integrity. Eur J Nutr. (2018) 58:1854. 10.1007/s00394-018-1854-3 30392135

[41] Nolasco EL Zanoni FL Nunes FP Ferreira SS Freitas LA Silva MC. Insulin modulates liver function in a type i diabetes rat model. Cellular physiology and biochemistry. Int J Exp Cell Physiol Biochem Apharmacol. (2015) 36:1467–79. 10.1159/000430311 26160428

[42] Ohira M Tanaka S Watanabe Y Nakamura S Oka R Yamaguchi T. Association of Plasma Xanthine oxidoreductase with arterial stiffness in Type 2 diabetes with liver dysfunction. Am J Med Sci. (2021) 363:242–50. 10.1016/j.amjms.2021.09.011 34619144

[43] Mohamed A. Aetiology and Presentation of Fatty Liver Diseases in Sudanese Patients. Khartoum: University of Khartoum (2005).

[44] Wada J Nakatsuka A. Mitochondrial dynamics and mitochondrial dysfunction in diabetes. Acta Med Okayama. (2016) 70:151. 10.18926/AMO/54413 27339203

[45] Herst PM Rowe MR Carson GM Berridge MV. Functional mitochondria in health and disease. Front Endocrinol. (2017) 8:296. 10.3389/fendo.2017.00296 29163365 PMC5675848

[46] Højlund K Mogensen M Sahlin K. Mitochondrial dysfunction in type 2 diabetes and obesity. Endocrinol Metabol Clin North America. (2008) 37:713–31. 10.1016/j.ecl.2008.06.006 18775360

[47] Ong SB Hausenloy DJ. Mitochondrial dynamics as a therapeutic target for treating cardiac diseases. Handb Exp Pharmacol. (2016) 2016:7. 10.1007/164_2016_7 27844171

[48] Friedman JR Lackner LL West M Dibenedetto JR Voeltz GK. Er tubules mark sites of mitochondrial division. Science. (2011) 334:358–62. 10.1126/science.1207385 21885730 PMC3366560

[49] Goldsmith R Joanisse DR Gallagher D Pavlovich K Shamoon E Leibel RL. Effects of experimental weight perturbation on skeletal muscle work efficiency, fuel utilization, and biochemistry in human subjects. Am J Physiol - Regulatory Inte Compar Physiol. (2010) 298:2009. 10.1152/ajpregu.00053.2009 19889869 PMC2806213

[50] Carlson LA Göran Ekelund L Fröberg SO. Concentration of triglycerides, phospholipids and glycogen in skeletal muscle and of free fatty acids and β-hydroxybutyric acid in blood in man in response to exercise. Eu J Clin Invest. (2018) 1:284–94. 10.1111/eci.1971.1.4.248 5549529

[51] Mori N Li G Fujiwara M Ishikawa S Arai T. Lipotoxicity observed at the early phase of obesity in cats fed on high-fat diet. Asian J Anim Vet Adv. (2014) 9:134–43. 10.3923/ajava.2014.134.143

[52] Turner N Bruce CR Beale SM Hoehn KL So T Rolph MS. Excess lipid availability increases mitochondrial fatty acid oxidative capacity in muscle: evidence against a role for reduced fatty acid oxidation in lipid-induced insulin resistance in rodents. Diabetes. (2007) 22:93. 10.2337/db07-0093 17519422

[53] Ortmeyer HK. In vivo insulin regulation of skeletal muscle glycogen synthase in calorie-restricted and in ad libitum–fed rhesus monkeys. J Nutri. (2001) 131:907S. 10.1093/jn/131.3.907S 11238784

[54] Meynial-Denis D Miri A Bielicki G Mignon M Renou JP Grizard J. Insulin-dependent glycogen synthesis is delayed in onset in the skeletal muscle of food-deprived aged rats. J Nutri Biochem. (2005) 16:150–4. 10.1016/j.jnutbio.2004.12.001 15741049

[55] Kowluru RA Mishra M. Therapeutic targets for altering mitochondrial dysfunction associated with diabetic retinopathy. Expert Opin Ther Targets. (2018) 22:233–45. 10.1080/14728222.2018.1439921 29436254 PMC6088375

[56] Chau MD Gao J Yang Q Wu Z Gromada J. Fibroblast growth factor 21 regulates energy metabolism by activating the ampk–sirt1–pgc-1α pathway. Proceed Nat Aca Sci USA. (2017). 107:2017. 10.1073/pnas.1006962107 20616029 PMC2906565

[57] Hao H Rutter J. The role of pas kinase in regulating energy metabolism. IUBMB Life. (2010) 60:204–9. 10.1002/iub.32 18344204

[58] Kannappan R Zhang EY Signore S Palano G Leri A. Regulates the myocardial recovery with diabetes aha scientific sessions. In: Conference: AHA Scientific Sessions 2014, Vol. 115. Chicago, IL (2014). p. e86–93.

[59] Liu J Liu JX Xu SN Quan JX Tian LM Guo Q. Association of p213s polymorphism of the l-selectin gene with type 2 diabetes and insulin resistance in chinese population. Gene. (2012) 509:286–90. 10.1016/j.gene.2012.07.086 22921892

[60] Terada Y Inoshita S Nakashima O Kuwahara M Sasaki S Marumo F. Cyclins and the cyclin-kinase system–their potential roles in nephrology. Nephrology, dialysis, transplantation : official publication of the European Dialysis and Transplant Association - European Renal Association. Nephrol Dial Transplant. (1998) 15:1913–6. 10.1093/ndt/13.8.1913 9719135

[61] Yang F Yi M Liu Y Wang Q Hu Y Deng H. Glutaredoxin-1 silencing induces cell senescence via p53/p21/p16 signaling axis. J Prot Res. (2018) 17:1091–100. 10.1021/acs.jproteome.7b00761 29356545

[62] Tang H Xu L Liang X Gao G. Low dose dinaciclib enhances doxorubicin-induced senescence in myeloma rpmi8226 cells by transformation of the p21 and p16 pathways. Oncol Lett. (2018) 16:6608–14. 10.3892/ol.2018.9474 30405800 PMC6202540

[63] Chiahsuan C Lee J Ravichandran R Fleming T Sureshbabu A. IGFBP2 protects against pulmonary fibrosis through inhibiting P21-mediated senescence. Cold Spring Harbor Labor. (2021). [*Preprint*]. 10.1101/2021.01.21.427684

[64] Van Deursen JM. The role of senescent cells in ageing. Nature. (2014) 509:436–9. 10.1038/nature13193 24848057 PMC4214092

